# A potential biomarker hsa-miR-200a-5p distinguishing between benign thyroid tumors with papillary hyperplasia and papillary thyroid carcinoma

**DOI:** 10.1371/journal.pone.0200290

**Published:** 2018-07-13

**Authors:** Xian Wang, Shan Huang, Xiaocan Li, Dongrui Jiang, Hongzhen Yu, Qiang Wu, Chaobing Gao, Zhengsheng Wu

**Affiliations:** 1 Department of Pathology, Anhui Medical University, Hefei, Anhui, China; 2 Department of Pathology, The Second Affiliated Hospital of Anhui Medical University, Hefei, China; 3 Department of Otorhinolaryngology, Head and Neck Surgery, The First Affiliated Hospital of Anhui Medical University, Hefei, China; Institute of Biochemistry and Biotechnology, TAIWAN

## Abstract

Papillary thyroid carcinoma (PTC) is the most common endocrine cancer with a significantly increase of the incidence recently. Several cytokines, such as thyroid peroxidase (TPO), cluster of differentiation 56 (CD56), Galectin-3, mesothelial cell (MC), cytokeratin 19 (CK19) and BRAF (B-raf) were recommended to be tested by immunohistochemistry (IHC) for a definitive diagnosis, but were still limited in clinical use because of their relative lower sensitivity and specificity. MicroRNA (miRNA), as a new molecular biomarkers, however, has not been reported yet so far. To address this, hsa-miR-200a-5p, a miRNA, was selected and detected in PTC patients by in situ hybrization with benign thyroid tumor with papillary hyperplasia as a control, and the differential expression of hsa-miR-200a-5p between fresh PTC tissues and control was detected by qRT-PCR. Expressive levels of cytokines of TPO, CD56, Galectin-3, MC, CK19 and B-raf were also detected by immunohistochemistry. The correlation was analyzed by SPSS software using Spearman methods. As expected, the hsa-miR-200a-5p expressive level was significantly increased in PTC patients, compared to that of control, and was consistent with that of TPO, CD56, Galectin-3, MC, CK19 and B-raf. In addition, expression of hsa-miR-200a-5p showed negative correlation to that of TPO (r_s_ = - 0.734; **: *P* < 0.01) and CD56 (r_s_ = - 0.570; **: *P* < 0.01), but positive correlation to that of Galectin-3 (r_s_ = 0.601; **: *P* < 0.01), MC (r_s_ = 0.508; **: *P* < 0.01), CK19 (r_s_ = 0.712; **: P < 0.01) and B-raf (r_s_ = 0.378; **: P < 0.01). PTC and papillary benign thyroid papillary hyperplasia are difficult to distinguish in morphology, so requiring immunohistochemistry to further differentiate the diagnosis, however, for the existing clinical common diagnostic marker for immunohistochemistry, the sensitivity and accuracy are low, it is easy to miss diagnosis. Therefore, there is an urgent need for a rapid and sensitive molecular marker. So miR-200a-5p can be used to assist in the diagnosis of PTC at the molecular level, and as a biomarker, can be effectively used to distinguish between PTC and benign thyroid tumor with papillary hyperplasia.

## Introduction

Thyroid carcinoma is the most common endocrine cancer with a significantly increase of the incidence in recent years, especially in young men [1–7]. According to the histogenesis and morphology, thyroid carcinomas can be classified into papillary thyroid carcinoma (PTC) [8], follicular carcinoma [9], medullary carcinoma [10] and undifferentiated carcinoma [11]. PTC is the most common thyroid malignant tumor, generally with an indolent clinical course, accounting for about 60% to 70% of total thyroid cancers, [12–14]. The overall 5-year relative survival rate has been reported as high as 97.5%, and only a small percentage of papillary carcinomas show aggressive clinical behavior [12–13, 15–18]. Typical PTC is characterized by papillary structures with characteristic nuclear morphology, such as glassy nuclei, nuclear grooves, and intranuclear inclusions. But it is difficult to distinguish from thyroid benign lesions, such as nodular goiter, Hashimoto's thyroiditis, and thyroid adenoma with papillary growth. At present, there are some markers for the differential diagnosis of PTC and benign thyroid tumor with papillary hyperplasia, such as CK19/Galectin-3/HBME1, but they are limited in clinical use because of their relative lower sensitivity and specificity. So it remains difficult in the differential diagnosis[19]. With the development of molecular biology and the emergence of various biomarkers, many researchers try to find new molecular biomarkers for early diagnosis and evaluation of prognosis of thyroid cancers.

MicroRNA (miRNA) are small non-corning RNA, approximately 18–22 nucleotide, and can post-transcriptionally regulate gene expression by binding to 3′-untranslated region of mRNAs, regulating target mRNAs transcript degradation or translational repression, and then extensively regulating biological functions, including tumorigenesis and development [20–23]. In addition, many researchers have reported the integrated genomic characterization, microRNA, gene expression and transcription factors signature of papillary thyroid carcinoma, and confirmed the correlation between PTC and microRNA [24–26]. Hsa-miR-200 family is a hot topic in recent years, which includes 5 members (miR-200a, miR-200b, miR-200c, miR-141 and miR-429) located on two different genomic clusters: one cluster including miR-200a, miR-200b and miR-429 on chromosome 1, and another cluster including miR-200c and miR-141 on chromosome 12[27–28]. Hsa-miR-200a, as one of its important members, has begun to attract much more attention since studies showed that hsa-miR-200a could inhibit the occurrence of renal cell carcinoma by inducing cell apoptosis through directly targeting SIRT1 [29–30]. It can regulate the endometrial cancer cell growth in vitro by targeting phosphatase and tensin homolog (PTEN) [31–32]. In addition, in tumorigenesis of colorectal cancer, hsa-miR-200a can target PTEN to promote colorectal cancer development. Chen *et al*, found that the low expression of hsa-miR-200a in hepatocellular carcinoma cells, and it could inhibit the proliferation, migration and invasion of hepatocellular carcinoma cells by targeting FOX2, suggesting that miR-200a might be used as a therapeutic molecule for liver cancer[33]. Furthermore, the miR-200 family can regulate the epithelial-mesenchymal transition induced by EGF/EGFR in anaplastic thyroid cancer cells [34]. A previous study by members of the research group found that the expression of miR-200 a was positively correlated with the degree of tumor differentiation in colorectal cancer, but not with age, gender, tumor size, tumor invasion depth, lymph node metastasis, and TNM stage[35]. miRNA precursors that generate two kinds of abundant miRNAs by instance such mature sequences are denoted the miR-5p (5’arm) and miR-3p (3’arm) [36]. For miR-200a-5p, one study found miR-200a-5p and suppresses the proliferation of human ovarian carcinoma cells by promoting p21 expression in a p53-independent manner[37]. Because the diagnose between benign and malignant thyroid tumors with papillary structure is easy to confuse, and our preliminary experiments showed that the expression of miR-200a-5p was higher in papillary thyroid carcinoma than in papillary thyroid tissue, so we want to explore whether miR-200a can be used as a new marker for the diagnosis of benign and malignant thyroid tumors with papillary structure. In our tests, the hsa-miR-200a-5p expressive level was significantly increased in PTC patients, consistent with that of TPO, CD56, Glectin-3, MC, CK19 and B-raf, which provides an experimental evidence for the possible diagnosis and treatment of PTC with hsa-miR-200a-5p.

## Materials and methods

### Specimens

In this study, 28 papillary benign thyroid tumors with papillary hyperplasia (as a control) and 40 thyroid carcinomas (PTC) patients were selected, which were surgical specimen at The Second Affiliated Hospitals of Anhui Medical University (Hefei, China) between 2015 and 2016. Patients who had been administered either chemotherapy or radiation therapy before surgery were excluded. The diagnoses were made according to the 2017 World Health Organization (WHO) classification of tumors, and were confirmed by permanent histology. The use of patient samples was approved by The Biomedical Ethics Committee of Anhui Medical University, with requirement of written informed consent from each patient (institutional review board-approved protocol number: 20180283).

### Immunohistochemistry (IHC)

The aforementioned papillary benign thyroid tumor with papillary hyperplasia and thyroid carcinoma tissues were fixed and sliced to perform IHC staining according to the manufacturer’s instruction. Endogenous peroxidase was inactivated by incubating the sections in 3% H_2_O_2_ for 30 min. The sections were subjected to sequential incubations with 10% normal goat serum in 0.01 M PBS for 30 min at room temperature, and then respectively incubated in mouse TPO antibody (MAB-0630, 1:200; Maxim biotechnology development Co., LTD, Fuzhou, China), mouse CD56 antibody (Kit-0028, 1:200; Maxim biotechnology development Co., LTD, Fuzhou, China), mouse Galectin-3 antibody (MAB-0572, 1:200; Maxim biotechnology development Co., LTD, Fuzhou, China), mouse MC antibody (MAB-0130, 1:200; Maxim biotechnology development Co., LTD, Fuzhou, China), mouse CK19 antibody (KIT-0030, 1:200; Maxim biotechnology development Co., LTD, Fuzhou, China) and mouse B-raf V600E antibody (790–4855, 1:500, Roche, USA) in PBS containing 0.3% Triton X-100 overnight at 4°C. The sections were washed three times with PBS for 5 min each and then incubated with peroxidase-conjugated goat anti-mouse IgG (1: 200; Zymed, South San Francisco, CA, USA) for 1 hrs at room temperature. Finally, the sections were developed with DAB in 0.1 M Tris-buffered saline (TBS) containing 0.001% H_2_O_2_ for 30 min. The sections were observed under a microscope (Olympus, Tokyo, Japan), and five specific areas in each region were captured, and 10% or more was used as a cut-off line for positive in the slide evaluation.

### In situ hybridization

The aforementioned tissues were fixed and sliced to perform in situ hybridization staining, according to the manufacturer’s instructions. The DNA sequence of the hybridization probe is 5′-TCCAG CACTG TCCGG TAAGA TG-3′. Slides were baked at 60°C oven for 2 hrs followed by de-waxing and hydration. Endogenous peroxidase was inactivated by incubating the sections in 3% H_2_O_2_ for 30 min. Added a total of 1 ml pepsase, which was diluted with 3% citric acid, and digested for 30 min at 37°C, and then washed with PBS for 3 times (5 min / time). Section was fixed by 1% paraformaldehyde / 0.1 M PBS (pH = 7.2–7.6) at room temperature for 10 min, including 1‰ DEPC, and washed by ddH_2_O for 3 times (5 min / time). Subsequently, added a total of 20 μl pre-hybridization solution, and incubated at 42°C for 2 hrs, and then hybridized with 20 μl pre-hybridization solution, and incubated at 42°C overnight. Then, sections were washed by 2×SSC solution at 37°C for 2 times (5 min / time), and then washed by 0.5×SSC solution at 37°C for 15 min, and also washed by 0.2×SSC solution at 37°C for 15 min. Dropwise add blocking solution, and incubated at 37°C for 30 min, and then added biotinylation mouse anti-digoxin, and incubated at 37°C for 60 min with PBS washing for 4 times (5 min / time). SABC was added drop by drop and incubated at 37°C for 20 min with PBS washing for 3 times (5 min / time), and then added biotinylation peroxidase, and incubated at 37°C for 20 min with PBS washing for 3 times (5 min / time). Developed in 50 μl DAB solution for 10 min, and washed with ddH_2_O followed by re-dyeing with hematoxylin. The sections were observed under a microscope (Olympus, Tokyo, Japan), and five specific areas in each region were captured, and10% or more was used as a cut-off line for positive in the slide evaluation.

### Quantitative real time polymerase chain reaction (qRT-PCR) assay

The total RNA of fresh tissue was extracted using the phenol-chloroform method, was reversely transcribed with a Reverse Transcription Kit (Takara, Japan) according to the manufacturers’ instructions. The reaction mixture, including 2 μl 5 ×RT buffer, 0.5μl RT Enzyme Mix, 0.5μl P-primer, 500 ng total RNA, RNase Free ddH_2_O up to 10 μl, was prepared and reacted at 37°C for 15 min, followed by 95°C for 5min, and then 4°C hold. Reverse transcription reactions were performed using qRT-PCR kit (Takara) according to the manufacturers’ instructions. The reaction mixture, including 10 μl of 2 × SYBR Premix Ex Taq Ⅱ, 0.8 μl of forword primer, 0.8 μl of reverse primer, 0.4 μl of ROX Reference DyeⅡ, 2 μl of cDNA and 6 μl of ddH_2_O, was prepared, and the qRT-PCR was performed according to the following program: one cycle of 95°C for 30 s; 40 cycles of 95°C for 5 s, 60°C for 34 s, and 4°C hold. Finally, the data was analyzed using the SDS 1.4 software (Applied Biosystems) based on 2^-△△Ct^, and histogram analysis was performed using the Origin 9.0 software.

### Statistical analysis

All statistical analyses were performed using SPSS software system for Windows (version 22.0; SPSS, Chicago, IL). Spearman co-efficient was calculated to determine the correlation between the expression of PTC and established biomarkers, such as hsa-miR-200a-5p, TPO, CD56, Glectin-3, MC, CK19 and B-raf. *p* < 0.05 and *p* < 0.01 were considered as significant differences and highly significant differences, respectively.

## Results

### The hsa-miR-200a-5p expressive level was significantly increased in papillary thyroid carcinoma patients

**Fig 1:**
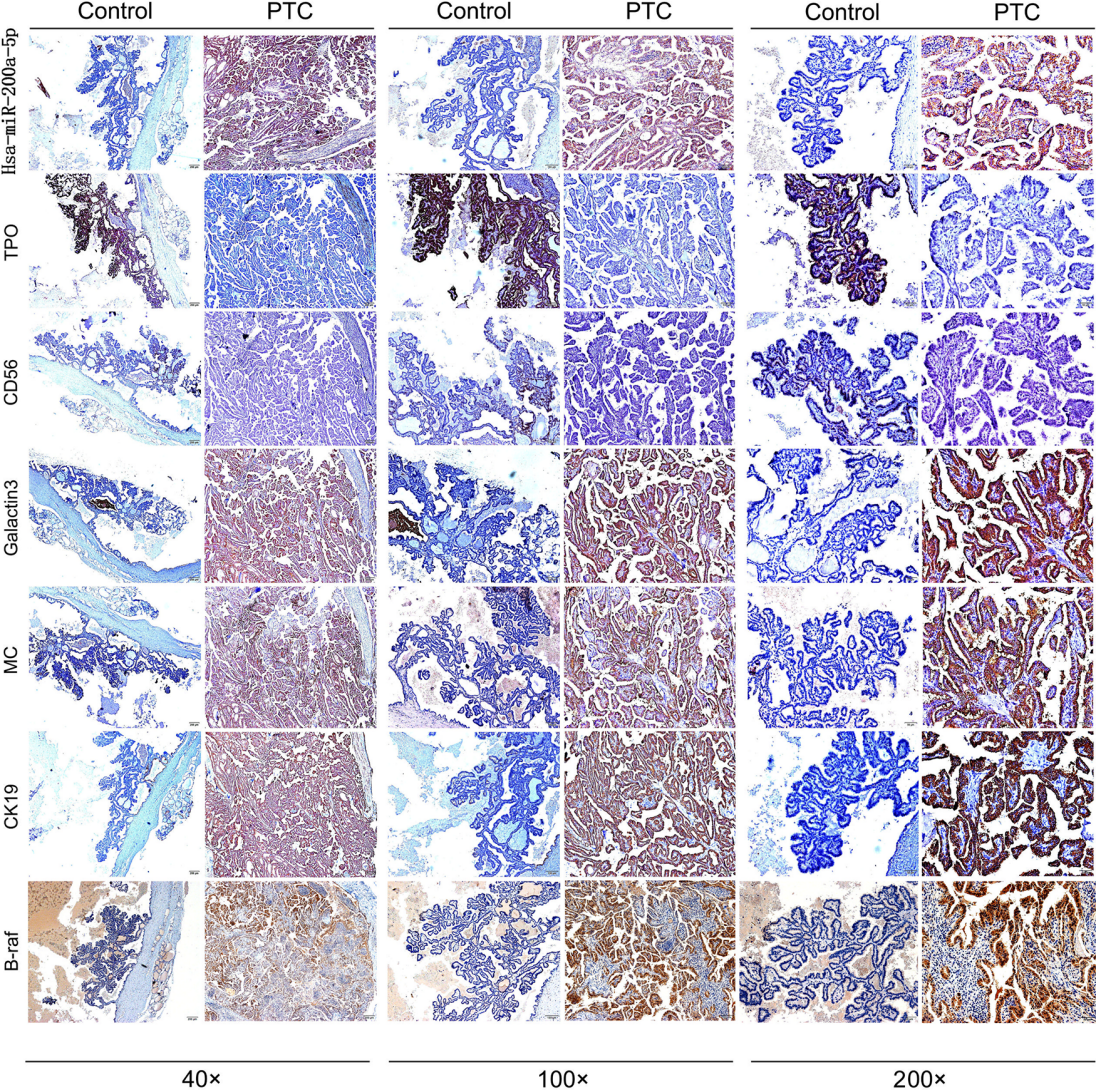
The assay of hsa-miR-200a-5p expressive level by in situ hybridization, and TPO, CD56, Galectin-3, MC, CK19 and B-raf expressive levels by immunohistochemistry. Compared to controls, the expressions of hsa-miR-200a-5p, Galectin-3, MC, CK19 and B-raf in PTC were higher while that of TPO and CD56 was lower.

As in Fig 1 and Table 1, when compared to control, the hsa-miR-200a-5p expressive level was significantly increased in PTC patients, consistent with that of Galectin-3, MC, CK19 and B-raf. However, the expressive level of TPO and CD56 was significantly decreased.

**Table 1:** The expressive levels of miR-200a-5p, TPO, CD56, Galectin-3, MC, CK19 and B-raf in benign thyroid tumors with papillary hyperplasia and papillary thyroid carcinoma.

Expressive levels		Tumor types	
		Benign thyroid tumor with papillary hyperplasia (n = 28)	Papillary thyroid carcinoma (n = 40)
miR-200a-5p	Positive, n (%)	5 (17.86)	35 (87.50)**
	Negative, n (%)	23 (82.14)	5 (12.50)
TPO	Positive, n (%)	28 (100)	3 (7.50)
	Negative, n (%)	0 (0.00)	37 (92.50)**
CD56	Positive, n (%)	24 (85.72)	0 (0.00)
	Negative, n (%)	4 (14.28)	40 (100)**
Galectin3	Positive, n (%)	3 (10.72)	40 (100)**
	Negative, n (%)	25 (89.28)	0 (0.00)
MC	Positive, n (%)	5 (17.86)	39 (97.50)**
	Negative, n (%)	23 (82.14)	1 (2.50)
CK19	Positive, n (%)	13 (46.43)	40 (100) **
	Negative, n (%)	15 (53.57)	0 (0.00)
B-raf	Positive, n (%)	0 (0.00)	32(80.00) **
	Negative, n (%)	28(100)	8(20.00)

Note

**, *P* < 0.01.

TPO, thyroid peroxidase

CD56, cluster of differentiation 56

MC, mesothelial cell

CK19, cytokeratin 19

B-raf, BRAF.

### The hsa-miR-200a-5p expressive level was significantly increased in papillary thyroid carcinoma patients fresh tissue

**Fig 2:**
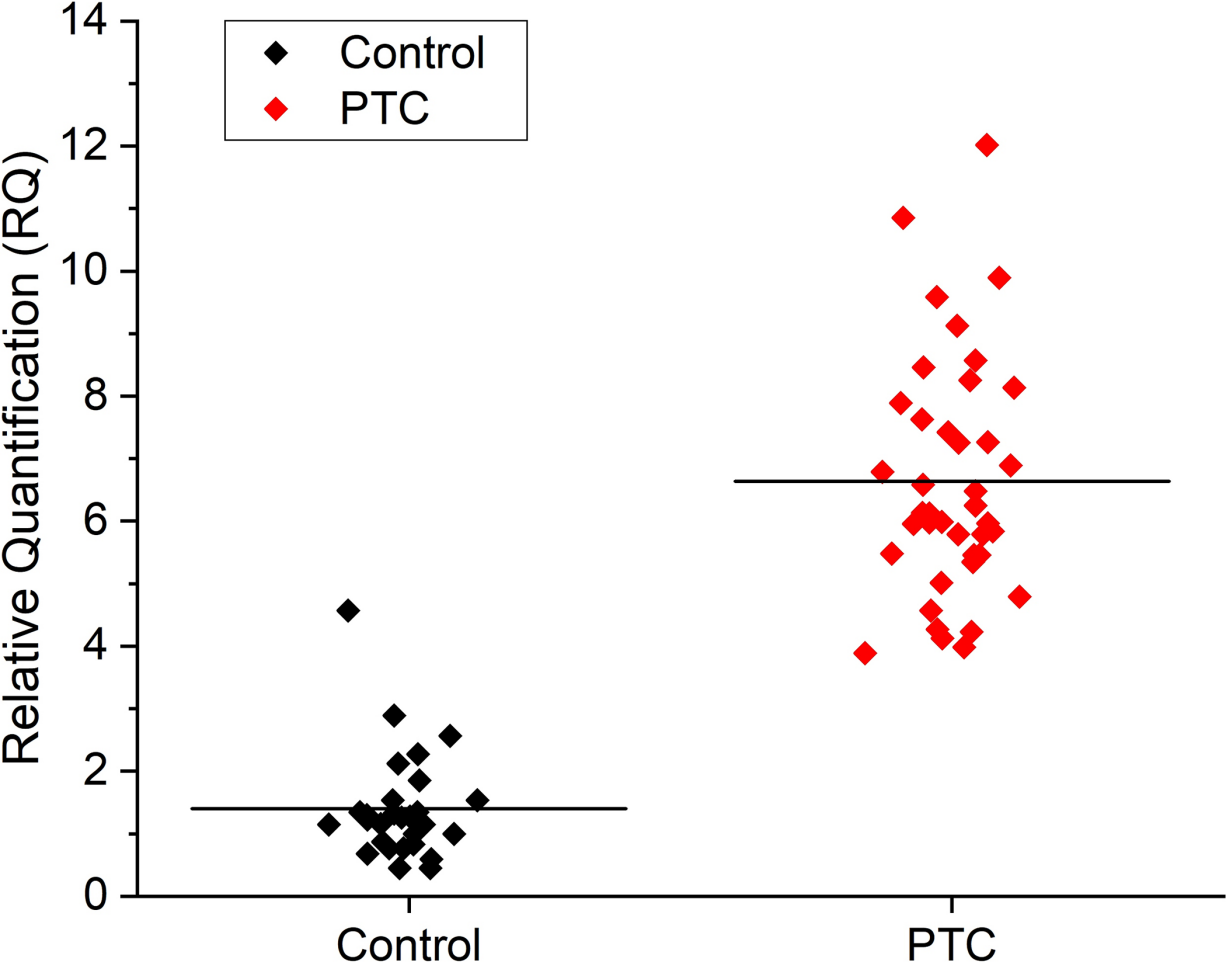
Expression level of hsa-miR-200a-5p in PTC by qRT-PCR. The expression level of hsa-miR-200a-5p in PTC fresh tissue was significantly increased compared with the control.

As exhibiting of Fig 2, the expression level of hsa-miR-200a-5p was significantly increased in fresh tissue of PTC patients (mean = 6.767, n = 40), when compared to that of control (mean = 1.402, n = 28) (**: p < 0.01).

### The hsa-miR-200a-5p expression showed positive correlation to that of galectin-3, MC, CK19 and B-raf, and negative correlation to that of TPO and CD56 in papillary thyroid carcinoma

In Table 2, by SPSS software using Spearman method for correlation analysis, the hsa-miR-200a-5p expression showed negative correlation to that of TPO and CD56, with coefficients of correlation (rho) -0.734 and -0.570 (**: *p* < 0.01) respectively, and showed positive correlation to that of Galectin-3, MC, CK19 and B-raf, with the coefficients of correlation (rho) 0.601, 0.508, 0.712 and 0.378 (**: *p* < 0.01), respectively.

**Table 2:** Correlation analysis of the expressions of has-miR-200a-5p to TPO, CD56, Galectin-3, MC, CK19 and B-raf in papillary thyroid carcinoma.

Index		n		TPO		CD56		Galectin-3		MC		CK19		B-raf	
				+	-	+	-	+	-	+	-	+	-	+	-
**miR-200a-5p**	**Benign thyroid tumor with papillary hyperplasia**	28	+	5	0	5	0	0	5	0	5	2	3	0	5
			-	23	0	19	4	3	20	5	18	11	12	0	23
	**Papillary thyroid carcinoma**	40	+	1	34**	0	35^##^	35^$$^	0	34^&&^	1	35^§§^	0	30^※※^	5
			-	2	3	0	5	5	0	5	0	5	0	2	3

Note: TPO, thyroid peroxidase; CD56, cluster of differentiation 56; MC, mesothelial cell; CK19, cytokeratin 19.

**: Correlated to miR-200a-5p. P < 0.01, r_s_ = -0.734

^##^: Correlated to miR-200a-5p. P < 0.01, r_s_ = -0.570

^$$^: Correlated to miR-200a-5p. P < 0.01, r_s_ = 0.601

^&&^: Correlated to miR-200a-5p. P < 0.01, r_s_ = 0.508

^§§^: Correlated to miR-200a-5p. P < 0.01, r_s_ = 0.712.

^※※^: Correlated to miR-200a-5p. P < 0.01, r_s_ = 0.378.

## Discussion

Here, we demonstrated that the expressive level of hsa-miR-200a-5p was significantly increased in the PTC, compared to that of control. It was negatively correlated to the expressions of TPO and CD56, with coefficients of correlation (rho) -0.734 and -0.570 (**: *p* < 0.01) respectively, while positively correlated to the Galectin-3, MC, CK19 and B-raf, with coefficients of correlation (rho) 0.601, 0.508, 0.712 and 0.378 (**: *p* < 0.01) respectively. In addition, for the existing clinical common diagnostic marker for immunohistochemistry, the sensitivity and accuracy are low, it is easy to miss diagnosis. Therefore, there is an urgent need for a rapid and sensitive molecular marker. Therefore, miR-200a-5p can be used to assist in the diagnosis of PTC at the molecular level, and as a biomarker, can be effectively used to distinguish between PTC and benign thyroid tumor with papillary hyperplasia.

Identification of new and sensitive biomarkers is still a priority to improve differential diagnosis of papillary thyroid carcinoma (PTC) and benign thyroid tumor. Although Galectin-3 and MC are frequently expressed in PTC [38], and TPO and CD56 are frequently expressed in benign thyroid tumor [39], the sensitivity of these markers remains unsatisfactory. For example, TPO is the key enzyme for the synthesis of thyroxin (T3, and T4), and plays an important role in the processes of activation of iodine, iodization of tyrosine residues, and coupling of iodinated tyrosine residues [40–41]. In recent years, scholars have begun to study the table of TPO in the transformation of thyroid adenopathy and explore its practical value, but the conclusion is still in dispute. The majority of reports indicate that TPO is in low form or lack of expression in thyroid malignant tumor [42], and TPO immunostaining has higher sensitivity and specificity to distinguish thyroid benign and malignant disease [43–44]. CD56 is a member of the immunoglobulin superfamily, and the significance of its expression on malignant tumors, such as small cell lung cancer [45], thyroid gland tumor [46] and prostate cancer is still being studied [47]. Similar to TPO, CD56 is in low form or lack of expression in PTC, but has a higher expression in normal thyroid tissue, such as follicular adenoma, nodular goiter and papillary hyperplasia. Galectin-3 is a β-galactose binding protein, which may participate the process of the cell growth, adhesion, inflammation, immune-regulation and apoptosis, and therefore associated with the formation and metastasis of many tumors, such as colon cancer and PTC [48–49]. In current application, Mesothelial cell (MC) is a mesothelioma related antigen, and has been used as an immunogen to produce monoclonal antibody. The MC was strongly expressed in mesothelioma and has been used in thyroid pathology diagnosis as well [50]. The high expression of MC in thyroid carcinoma appears to be a good marker for thyroid papillary carcinoma. BRAF somatic mutations, was the most extensively investigated molecular markers and common genetic alterations in PTC [51]. Many studies have found that higher expression of Braf mutant protein can predict aggressive tumor behavior in PTC, and BRAF V600E IHC has high practical value for the detection of the BRAF V600E mutation in primary and metastatic PTC [52–53]. Furthermore, the results of several studies have challenged the notion that the BRAFV600E mutation is a valuable prognostic marker. The sensitivity and specificity of TPO, CD56, Galectin-3, MC, CK19 and B-raf, as existing common biomarkers, were still too limited and unsatisfactory to be widely used in clinical diagnosis.

miRNAs are no-coding RNAs with a length of 18–24 nt, and could be down-regulated the target gene expression at transcriptional and translational levels. As in several previous documented studies, some miRNAs might be involved in the regulation of biological processes that are important for cancer cells, which indirectly promote the functions of oncogene and tumor suppressors [54–55]. For instance, hsa-miR-200a-5p, as an important members of miR-200 family, has been paid more attention recently. Many studies showed that the high expression of hsa-miR-200a-5p was present in a variety of cancers, including colorectal cancer, pancreatic cancer, esophageal adenocarcinoma, but remains unclear in thyroid neoplsam. Herein, we evaluated the hsa-miR-200a-5p expression in papillary thyroid carcinoma and benign thyroid tumor with papillary hyperplasia using formalin-fixed paraffin-embedded samples. Our data demonstrated that the expression of hsa-miR-200a-5p increased significantly in PTC. In comparison with other established biomarkers for PTC, the expression rate of hsa-miR-200a-5p was higher than that of Galectin-3 and MC, and therefore can be in differential diagnosis between PTC and benign thyroid tumors. In addition, according to the correlation, we found that has-miR-200a-5p was negatively correlated to TPO (r_s_ = - 0.734; **: *P* < 0.01) and CD56 (r_s_ = - 0.570; **: *P* < 0.01), while hsa-miR-200a-5p was positively correlated to Galectin-3 (r_s_ = 0.601; **: *P* < 0.01), MC (r_s_ = 0.508; **: *P* < 0.01), CK19 (r_s_ = 0.712; **: P < 0.01) and B-raf (r_s_ = 0.378; **: P < 0.01). The above results indicated that hsa-miR-200a-5p, as a biomarker, can be effectively used to distinguish between papillary thyroid carcinoma and benign thyroid tumor with papillary hyperplasia.

In conclusion, this study firstly demonstrated that hsa-miR-200a-5p is a sensitive and specific biomarker for PTC with potential clinical application in the differential diagnosis between benign and malignant thyroid tumors, as well as basic and clinical-oriented researches. However, the mechanism is not clear. In the future, we will focus on these studies, and strengthen the proof of hsa-miR-200a-5p as a new biomarker to distinguish between papillary thyroid carcinoma and benign thyroid tumor with papillary hyperplasia, and other clinical diagnoses.
